# Validation and Factor Structure Analysis of the Polish Version of the Somatosensory Amplification Scale (SSAS-PL) in Clinical and Non-Clinical Samples

**DOI:** 10.3390/jcm14144846

**Published:** 2025-07-08

**Authors:** Krystian Konieczny, Karol Karasiewicz, Karolina Rachubińska, Krzysztof Wietrzyński, Mateusz Wojtczak

**Affiliations:** 1 Department of Clinical Psychology and Psychoprophylaxis, Institute of Psychology, University of Szczecin, 71-017 Szczecin, Poland; 2 Department Social and Developmental Psychology, Institute of Psychology, University of Szczecin, 71-017 Szczecin, Poland; 3 Department of Health Psychology, Pomeranian Medical University in Szczecin, 71-460 Szczecin, Poland; 4 Institute of Psychology, Cardinal Stefan Wyszynski University, 01-938 Warsaw, Poland

**Keywords:** somatosensory amplification, psychosomatics, validation, psychometry

## Abstract

**Objectives:** The aim of this study was to validate the Polish version of the Somatosensory Amplification Scale (SSAS-PL) and examine its psychometric properties in clinical and non-clinical samples. **Methods**: The study included 1128 participants (711 healthy adults, 194 cardiac patients, 223 psychiatric patients). The analyses were categorized into exploratory and confirmatory phases. Exploratory analyses were conducted on a randomly selected sample that comprised 60% of the study participants (training sample) to estimate the reliability (Cronbach’s alpha) and factorial validity (EFA with varimax rotation). Confirmatory analyses were performed on an independent (test) sample that represented 40% of the total sample size to facilitate the cross-validation of the factor structure (CFA) and to assess the convergent and discriminant validities (using the HTMT method) in relation to health anxiety (SHAI) and psychopathological symptoms (KOFF-58). Additionally, measurement invariance was examined with respect to gender (female vs. male) and health status (healthy vs. clinical). **Results:** The SSAS-PL demonstrated good internal consistency (α = 0.75–0.78) after removing item 1. A one-factor structure showed the best fit and theoretical interpretability. The measurement invariance was supported across clinical groups. The SSAS-PL showed convergent validity with the measures of somatic symptoms, anxiety, and health anxiety. It demonstrated discriminant validity from other psychopathology measures. **Conclusions:** The SSAS-PL was a reliable and valid measure of somatosensory amplification in the Polish population. Its unidimensional structure aligned with most cross-cultural adaptations. The scale may be useful for assessing somatosensory amplification in both research and clinical settings in Poland. Further research on its utility in specific clinical populations is warranted.

## 1. Introduction

Somatosensory amplification (SSA) is characterized by increased perception and concern regarding normal bodily sensations [1]. It is associated with heightened reactivity to both external and internal stimuli, leading to their interpretation as potential threats to bodily integrity [2]. Understanding SSA is crucial for comprehending the development and persistence of somatic symptoms that are not attributable to somatic diseases [3]. The three principal theoretical components of somatosensory amplification include (1) hypervigilance, which involves excessive alertness and heightened attention to bodily sensations; (2) a propensity to selectively focus on somatic and visceral sensations that are infrequent or of low intensity; and (3) a tendency to interpret bodily sensations as more alarming and threatening than they actually are [1]. The Somatosensory Amplification Scale (SSAS) was developed to measure SSA [1].

The findings of a 2020 meta-analysis suggest an association between SSA, somatic symptom disorder (SSD), anxiety disorders (ADs), and health anxiety (IAD). In the context of SSD and IAD, the primary influence on SSA may not be enhanced precision in perceiving interoceptive stimuli, but rather a bias in their interpretation, as influenced by cognitive factors, such as an anxiety bias [4]. One potential mechanism contributing to the observed increase in SSA is catastrophizing [5]. Individuals with anxiety disorders often interpret ambiguous bodily signals as pathological indicators. Other studies [6] have identified a correlation between SSA and trait anxiety and body awareness, which, according to the authors, aligns with research findings on the P300 potential magnitude, suggesting that SSA involves long-latency cognitive processing rather than short-latency interoceptive sensitivity [7]. Additionally, there are reports of a connection between SSA and generalized anxiety disorder (GAD) and alexithymia (e.g., ref. [8]), with a substantial body of research focusing on the relationship between SSA and hypochondria, given the observed comorbidity of these conditions [9,10,11,12,13].

The research conducted by Nakao et al. demonstrated a significant association between somatosensory amplification (SSA) and specific components of alexithymia, particularly the difficulty in identifying and describing feelings. These associations persisted even when controlling for variables such as age, gender, clinical group, and levels of anxiety and depression. Notably, the third component of alexithymia, external thinking orientation, did not exhibit any association with SSA, indicating that only certain facets of alexithymia contribute to an increased perception of bodily sensations. Individuals with impairments in recognizing and expressing emotions may be more susceptible to overestimating their somatic sensations, thereby facilitating the development of psychosomatic symptoms [14]. Research has shown that the Somatosensory Amplification Scale (SSAS) is a valuable tool for distinguishing between somatic symptom disorders and anxiety-related conditions. It aids in determining the extent to which symptoms are due to heightened perception as opposed to anxiety disorders. Individuals with somatic disorders tend to have higher SSAS scores than those with anxiety disorders [15]. Moreover, there are notable differences in the localization of somatic symptoms. Symptoms linked to anxiety disorders are mainly found in the cardiovascular and respiratory systems, while those associated with somatic disorders are dispersed across various non-specific regions of the body. Additionally, Nakao and Barsky [16] found that individuals with elevated SSAS scores are more likely to develop somatic disorders compared with those with typical anxiety symptoms.

A three-year longitudinal study has substantiated that SSA is a relatively stable psychological factor contributing to the persistence of somatic symptoms and diminished mental and physical functioning in patients with persistent physical symptoms (PPSs). Throughout the observation period, higher SSA scores were significantly correlated with increased symptom severity and decreased functioning, thereby distinguishing SSA from a time-varying symptom focus [17].

Individuals experiencing migraines exhibit elevated levels of somatosensory amplification (SSA) compared with their healthy counterparts. Elevated SSA scores are associated with an increased frequency of migraine episodes and heightened migraine-related disability (MIDAS), underscoring the significance of SSA as a factor that exacerbates symptoms [18]. Furthermore, SSA is correlated with anxiety and stress levels, suggesting that the overinterpretation of bodily sensations may mediate the relationship between psychological stress and the severity of somatic symptoms. Consequently, increased amplification may contribute not only to subjective distress but also to diminished functional capacity [19].

In a cohort of patients diagnosed with noncardiac chest pain (NCCP), the Somatosensory Amplification Scale (SSAS) scores were markedly elevated compared with those in the control group. Within the NCCP cohort, female patients exhibited higher levels of somatosensory amplification than their male counterparts, a pattern that was not observed in the control group. These patients demonstrate increased perception and focus on bodily signals, which may result in the misinterpretation of these signals as threatening. The significance of somatosensory amplification in the etiology and persistence of NCCP symptoms is underscored, along with the more frequent utilization of cardiac care services, particularly among female patients [18].

A study that examined patients who experienced mild heart palpitations revealed significantly elevated SSA scores despite the absence of an organic etiology. These findings were associated with increased anxiety (BAI score) and depression (BDI score). However, the regression analysis indicated that only anxiety levels served as an independent predictor of symptoms, while SSA did not achieve statistical significance. This implies that SSA functions as a mediator of subjective symptom expression rather than a primary mechanism underlying this phenomenon [20].

The research conducted by Sayar et al. examined the effect of antidepressant therapy on SSA levels in individuals diagnosed with both depression and fibromyalgia. Within the cohort that experienced depression, notable decreases in SSA levels were recorded after 6 and 12 weeks of treatment, which corresponded with reductions in the symptoms of depression and anxiety [21].

The Somatosensory Amplification Scale is the sole instrument available to assess somatosensory amplification. The original version comprised 10 items, with responses recorded on a 5-point Likert scale that ranged from 1 (strongly disagree) to 5 (strongly agree).

The Somatosensory Amplification Scale has been adapted to several languages, including French [22], Spanish [23], Chinese [24], Japanese [25], Korean [26], Turkish [27], Farsi [28], and Hungarian [29].

Most adaptations exhibit robust psychometric properties and typically endorse a single-factor questionnaire. However, factor analyses were conducted for models with one, two, and three factors in the referenced adaptations. In certain instances, the distribution of items facilitates the differentiation between exteroceptive and interoceptive factors. It is noteworthy that not all adapted versions preserved the original 10-item scale format.

The objective of this study was to assess the psychometric properties of the Somatosensory Amplification Scale (SSAS-PL) in a Polish cohort comprising healthy adults and patients with cardiac or psychiatric conditions. The study employed a three-stage analytical approach: (1) an exploratory analysis conducted on a subgroup of healthy adults (*n* = 426, which represented 59.9% of the healthy adult sample), (2) a confirmatory analysis performed on a sample of 285 healthy adults (which constituted 41.1% of the healthy adult sample), and (3) a measurement invariance analysis that involved a sample of cardiology and psychiatric patients (*n* = 417).

## 2. Materials and Methods

### 2.1. Study Procedure

Initially, consent was obtained from A. Barsky to adapt the SSAS to the Polish conditions. Subsequently, approval was obtained from the Bioethics Committee of the Institute of Psychology, University of Szczecin. The original American version of the SSAS was then translated by three independent translators, all of whom were native speakers with expertise in scientific texts. These translations were assessed by three judges for their content and linguistic correctness. The judges, who were psychologists with clinical experience, included two individuals who were researchers. Consequently, the most suitable translation for each test item was selected, which resulted in the use of a version of the test in the subsequent study phases. This study adhered to the ethical principles outlined in the Declaration of Helsinki. Each participant provided written consent to participate and was afforded the opportunity to ask questions and receive responses from a psychologist or researcher conducting the study.

The collected data were analyzed statistically to ascertain the psychometric properties of the questionnaire.

### 2.2. Participants

The study was conducted with a total of *N* = 1128 participants, which comprised *n* = 417 individuals in both clinical groups, which represented 37% of the sample, and *n* = 711 individuals in the healthy group, which accounted for 63% of the sample. The sample included *n* = 705 women (62.5%), *n* = 415 men (36.8%), and *n* = 8 individuals who identified with another sex (0.7%). The participants ranged in age from 18 to 92 years, with a mean age (M) of 42.9 years, standard deviation (SD) of 18.3, and median (MD) age of 43 years. A comprehensive description and characteristics of the study sample are presented in Table 1.

Within the sample of healthy individuals, the participants were randomly allocated into two subgroups: a training group (*n* = 426, 59.9%) and a test group (*n* = 285, 40.1%). In subsequent stages, data from the training subgroup was utilized to estimate the psychometric properties of the SSAS through the exploratory analyses, while data from the test subgroup was employed to assess the goodness of fit of the models in the confirmatory analyses. The comparative results presented in Table 2 substantiated the assumption that the training and test subgroups did not differ significantly in terms of age, gender, education, or place of residence, thereby confirming their parallel nature.

Validation studies were conducted using two sampling methods: purposive sampling, which involved a cohort of cardiology patients, and snowball sampling, which encompassed a general group and a group of psychiatric patients. All the study cohorts were assessed using the paper-and-pencil method. The investigation involving cardiology patients was conducted in healthcare facilities located in the West Pomeranian Vovoidship, Poland. During their visit to the facility, the patients were initially qualified for participation by a physician who considered factors such as the cognitive ability to complete the questionnaire and diagnose cardiac conditions. The psychiatric patient cohort was assembled using the snowball method, which leveraged the relationships between the study participants. Individuals that resided in closed psychiatric facilities or psychiatric day wards were excluded from this study. The participants in this group were stable. Inclusion in the study was based on a self-reported mental health status and previous psychiatric treatment. The psychiatric patient sample comprised individuals who reported undergoing psychiatric treatment but not cardiological treatment. This group also included individuals who reported drug use or alcohol consumption and were receiving psychiatric treatment but excluded those who reported alcohol consumption or drug use without undergoing psychiatric treatment.

Healthy adults were selected using snowball sampling. The cohort excluded individuals who reported drug use, psychiatric treatment, psychotherapy, or cardiovascular conditions (e.g., myocardial infarction, congenital heart defects, implanted bypasses, implanted cardioverter defibrillators, previous cardiac surgery, hypertension, and stroke), individuals under 18 years of age, and those who were dependent.

During the data analysis phase, responses from individuals who completed the questionnaires in an unreliable manner, specifically those whose responses significantly deviated from the norm, as determined by the Mahalanobis distance method, were excluded from further consideration. Consequently, 102 observations, which represented 8.9% of the total respondents, were omitted from the analysis.

### 2.3. Measures

The following instruments were used to assess the psychometric properties of the Somatosensory Amplification Scale.

**The Somatosensory Amplification Scale in the Polish experimental version** comprised 10 items that linguistically corresponded to those in the original English version. The responses were recorded on a 5-point Likert scale that ranged from 1 to 5 [1].

**The Short Health Anxiety Inventory (SHAI**)—The instrument in question is an 18-item self-report measure of health anxiety, originally developed by Salkovskis et al. [30] and subsequently adapted for the Polish population by Kocjan [31]. The participants responded using a 4-point Likert scale, with options that ranged from 0 (no symptoms) to 3 (very severe symptoms). The Polish adaptation has demonstrated reliability, with a Cronbach’s α of 0.91 in clinical samples and 0.92 in healthy samples.

**Kwestionariusz Ogólnej Oceny Funkcjonowania (KOOF-58) [ang. The General Functioning Questionnaire (GFQ-58)]**—The Polish instrument developed by Styła and Kowalski was employed to assess psychopathological symptoms across 13 distinct areas, as delineated by the ICD-10 and DSM-IV-TR. These areas were the (1) poor functioning at work and home scale, (2) lack of entertainment scale, (3) poor relationships scale, (4) cognitive disorders scale, (5) addictions scale, (6) productive symptoms scale, (7) depressive disorders scale, (8) manic disorders scale, (9) anxiety disorders scale, (10) eating disorders scale, (11) sleep disorders scale, (12) sexual disorders scale, and (13) somatic symptoms scale. The KOOF-58 demonstrates reliability characterized by Cronbach’s α, ranging from 0.89 to 0.92. The selection of this tool was justified by (a) its comprehensive measurement of psychopathological symptoms coupled with the relatively robust psychometric properties of the entire questionnaire and its scales, and (b) its ambiguous legal and psychometric status, as well as the potential for utilizing the SCL-90 in scientific research within the Polish language context [32].

**Sociodemographic survey**—An original survey was conducted to gather data on the participants’ age, sex, health status, history of illnesses, place of residence, and substance use. 

### 2.4. Statistical Analyses

In a sample of healthy subjects, exploratory factor analysis (EFA) with varimax rotation assuming an orthogonal factor solution was conducted on a randomly selected subset that comprised 60% of the respondents, referred to as the training sample. This analysis involved flexible adjustment of both one- and two-factor structures to describe somatosensory amplification within a healthy adult population. Subsequently, confirmatory factor analyses (CFAs) were performed on a separate random subset of 40% of respondents, designated as the test sample, which was independent of the training sample. The assumptions of multivariate normality were checked using the Mardia test. These analyses evaluated the fit of the single- and two-factor models, as estimated in the training sample, and considered models from other cultural contexts, including Polish, French, Iranian, and Chinese populations, to assess their goodness of fit in describing the construct under investigation among healthy Polish adults. Following this, a measurement invariance analysis was conducted for the model selected in the previous stage within a population of cardiology and psychiatric patients. Finally, convergent and discriminant validities were assessed by correlating the SSAS measurement results with those of the SHAI and KOFF-58 scales in a sample that comprised both healthy individuals and patients, irrespective of their health status.

## 3. Results

### 3.1. Reliability

A reliability analysis was conducted using Cronbach’s alpha coefficients. Within the subgroup of healthy adults (*n* = 426), the average inter-item correlation (average r), signal-to-noise ratio (S/N), and mean scale score and standard deviation were computed. We posited that a Cronbach’s alpha (α) greater than 0.70 and an average inter-item correlation (r) exceeding 0.25 indicate high reliability and internal consistency of the scale, while elevated signal-to-noise ratio (S/N) values corroborate the presumption of enhanced measurement precision. The analysis was performed in the test group. Table 3 presents the results of these analyses.

To assess the reliability, specifically the internal consistency, Cronbach’s α analysis was conducted on the one-dimensional solution of the SSAS measurement results.

The findings from the reliability analysis substantiated the assumption that the SSAS demonstrated reliability [α > 0.75] and internal consistency [r > 0.23], with the reliability index being comparable across both the training and test samples. A comprehensive examination of the discriminative power of individual test items indicated that within a population of healthy adults, item 1 (ang. “When someone coughs near me, I also start coughing”/pol. “Gdy ktoś przy mnie kaszle, mnie też dopada kaszel”) exhibited lower discriminative power relative to the other test items. Item 1 seemed to inadequately focus on individual bodily sensations, or, in the context of the Polish population, the observation of another person’s symptoms may not effectively correspond with the construct of somatosensory amplification.

Upon its exclusion from the training sample, the reliability coefficient increased from 0.75 to 0.77, the average correlation between items rose from 0.23 to 0.27, and the S/N ratio improved from 2.86 to 3.37. Detailed item-level reliability metrics for the training sample are presented in Table 4. Consequently, we recommend removing this test item from the amplification measurement model.

In the test subgroup, the results of the discriminant power analysis were similar (α = 0.78, mean r = 0.28, S/N = 3.54). Consequently, item 1 was excluded from the overall scale score in the subsequent analyses. It is recommended that future studies consider treating this item as a buffer.

A comprehensive review of the indicators for individual test items indicated that item 1 exhibited a notably low discriminatory power. Upon exclusion, there was a significant increase in both the Cronbach’s alpha reliability coefficient and the average correlation between items, as well as the signal-to-noise ratio (S/N). This finding implies that the test item in question possessed limited discriminatory capacity in assessing the construct under investigation across both training and test samples. Consequently, this test item should be excluded from subsequent analyses.

The analysis results indicate that while the overall reliability of the SSAS in the general population was satisfactory (α > 0.70), and the measurement of the first factor (MR1) extracted in the exploratory analysis was similarly reliable, the measurement of the second factor (MR2) was unsatisfactory in terms of the reliability (α < 0.60). This suggests that within the general population, only the measurement of the first factor was sufficiently accurate and consistent for describing somatosensory amplification. Assuming a two-factor model for interpreting the SAS results, it was observed that the reliability indicators for both factors were satisfactory or sufficient (α > 0.60). This indicates that the two-factor solution, despite having significantly lower factor validity than the three-factor solution, was characterized by a higher internal consistency, that is, reliability. Additionally, the reliabilities of the three-, two-, and one-factor models for the SSAS were analyzed without the SSAS1 item, in case it proved beneficial to exclude this item from the calculation results. The detailed results of this internal consistency analysis are presented in Table 5. In this subsequent step, item 1 was excluded from the scale to examine how its removal affects the internal consistency of the factor models. The same analysis was conducted on the indicators for the three-factor model (recommended based on the EFA), the two-factor model (which appeared to be just sufficient), and the one-factor model characteristic of other adaptations. The detailed results of this follow-up reliability analysis are presented in Table 6.

In conclusion, the reliability analysis indicated that the SSAS demonstrated satisfactory reliability and internal consistency in a population of healthy adults. In the final version, it is recommended to exclude responses to item 1 from the analysis of the measurement results.

### 3.2. Factor Accuracy

To ascertain the factor structure, exploratory factor analysis (EFA) was conducted on the data from the training sample. This analysis evaluated the fits of both the one- and two-factor models to the data in alignment with theoretical premises that propose either a one- or two-factor structure of somatosensory amplification. Exteroceptive and interoceptive amplifications were differentiated in the two-factor model. The validity of the single-factor model was assessed in relation to research findings suggesting that in most cultures or countries (e.g., France, China, Iran, and the original American version), the SSAS model typically adopts a single-factor structure. The model accuracy was determined by comparing the RMSEA, CFI, and BIC for the one- and two-factor models identified in the EFA. The fit indices for both models are summarized in Table 7.

**Figure 1:**
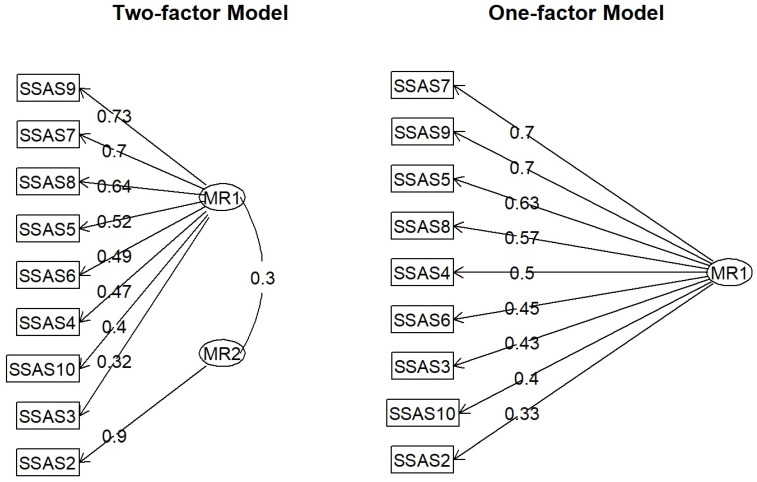
Path diagram of EFA results.

The findings corroborate the hypothesis that the two-factor model exhibited superior fit indices compared with the one-factor model [χ^2^(8) = 70.5548839, *p* < 0.001]. Nonetheless, the fit of the one-factor model within the training sample was deemed “just sufficient,” indicating its potential utility in measuring the construct under investigation. In summary, the analysis suggested a superior fit for the two-factor model relative to the one-factor model. However, a one-factor model is a viable option. A detailed examination of the model structure (see Figure 1) indicated that theoretically, the one-factor model was more appropriate for describing the SSAS results in the Polish version. An analysis of the results did not provide sufficient justification to endorse the two-factor model. It is imperative to examine the structure of factor loadings, as depicted in Figure 1.

The factor structure analysis results indicate that the two-factor model presented challenges in terms of interpretability within the theoretical framework. Specifically, the interpretation of the second factor (MR2), which was represented by a single test item, was difficult. Consequently, the single-factor solution was deemed to fit the data adequately and was theoretically robust for describing the outcomes of the SSAS scale measurement.

### 3.3. Verification of the Psychometric Properties of the SSAS Scale

The heterotrait–monotrait (HTMT) method [33] was employed to assess the convergent and discriminant validities. This approach, utilizing the SEM structural model, facilitates the estimation of not only the Cronbach’s α internal consistency index but also the Average Variance Explained (AVE), Average Shared Variance (ASV), and Maximum Shared Variance (MSV). A high AVE value signifies strong factorial validity, whereas a high MSV value indicates a robust convergent validity. Conversely, a high discriminant validity is demonstrated when the ASV value is lower than the AVE, suggesting that the variance shared with another construct is less than the variance of the scale itself. The convergent and discriminant validities were estimated in the context of health anxiety (SHAI) and the severity of psychopathological symptoms (KOFF-58). The results of this analysis are presented in Table 8.

The results of the convergent and discriminant analyses of SSAS in relation to SHAI and KOFF-58 demonstrated satisfactory or nearly satisfactory factorial validity for both the single-factor solution (nine items) and the two-factor solution (eight items), along with a significant differential validity (MSV < AVE) for each solution.

The AVE index exceeded 0.30, and the ASV index was lower than the AVE, indicating high levels of factor and differential validities for the SSAS scale in both the one-factor and two-factor models. In conclusion, the SSAS effectively described the outcomes of the nine test items using one or two differentiation factors, which were distinctly different from the results of the scales that measured the health anxiety and psychopathological symptoms.

Upon conducting the analyses, it was observed that the SSAS score was strongly correlated with somatic symptoms, anxiety disorders, and cognitive disorders. Conversely, the weakest negative correlations were identified with the addiction, lack of entertainment, and poor functioning at work and home scales. A comprehensive description of the SSA correlates is provided in Table 9.

### 3.4. The Structure of SSAS in Poland and Other Cultures

The literature presents several notable adaptations of the SSAS across various cultural contexts, suggesting either a one- or two-dimensional framework for assessing somatosensory amplification. Notably, discrepancies exist between these adaptations concerning the configuration of two-factor solutions. This study examined whether, within the Polish population, particularly among cardiology and psychiatric patients, the one- or two-factor model identified for the healthy adult population was more appropriately aligned with the data than the models proposed by other SSAS scale adaptations. To address this, a series of confirmatory factor analyses (CFAs) was performed, which considered different factor structures of the SSAS scale measurement, specifically a one- and two-factor structure in the Polish, Chinese, French, and Iranian adaptations. These models were subsequently evaluated based on their fit to the data obtained from a clinical sample of cardiology and psychiatric patients. The cross-cultural fit indices for the clinical population are summarized in Table 10.

The analysis results suggest that the two-dimensional model describing the SSAS scale outcomes, as identified in the population of healthy Polish adults, was equally applicable to data from the samples of cardiology and psychiatric patients. This model aligned with those proposed in the French and Chinese adaptations. However, the model proposed by the authors of the Iranian adaptation appeared to be marginally more suitable for the data than the model that described SSAS in the healthy adult population. In conclusion, the single-factor model (which comprised the sum of items 2 to 10) was likely to be a satisfactory model for describing SSAS scale outcomes in the clinical population of cardiology and psychiatry patients and showed no significant deviation from the models proposed in other cultural contexts (Iranian, Chinese, or French). Therefore, in terms of cultural appropriateness, it is recommended to employ a unidimensional model to describe the SSAS scale outcomes in cardiology and psychiatric patients.

### 3.5. Measurement Invariance of the SSAS Scale in a Clinical Population Depending on the Type of Health Disorder

To evaluate the hypothesis that the SSAS measurement model, whether one-dimensional or two-dimensional, maintains consistent accuracy across clinical populations irrespective of the specific health disorder, measurement invariance analysis was performed. This analysis involved a sample of cardiology and psychiatric patients and assessed changes in the model fit under the assumptions of (a) equality of factor loadings (weak/metric invariance); (b) equality of loadings and intercepts (strong/scalar invariance); and (c) equality of loadings, intercepts, and latent factor means (latent means invariance). The results of the invariance analysis are presented in Table 11.

The findings of the analysis indicate robust measurement invariance of the SSAS model within the patient population, contingent on the type of health disorder. Although the Δχ^2^ test revealed a statistically significant (*p* < 0.001) difference between the configural invariance model and the metric invariance model (weak invariance), this difference was minimal in terms of the effect size. This was evidenced by the negligible changes in the global fit indices of the models to the data, particularly the RMSEA and CFI, which remained below 0.03 for individual levels of equivalence. Consequently, it can be inferred that the one-dimensional SSAS measurement model was equivalent (invariant) across the cardiology and psychiatric patients, irrespective of the type of health disorder. Invariance was assessed for two types of disease: psychiatric and somatic (cardiological). This should be regarded as the initial phase of research, and it is not feasible to generalize invariance to other types of diseases, such as psychosomatic disorders.

## 4. Discussion

This study offered a thorough validation of the Polish version of the Somatosensory Amplification Scale (SSAS-PL) by examining its psychometric properties within a diverse cohort that comprised both healthy individuals and those with cardiac and psychiatric disorders. These findings yield significant insights into the reliability, factor structure, validity, and measurement invariance of the SSAS-PL, which are crucial for its prospective application in scientific research and clinical practice in Poland.

### 4.1. Internal Reliability

The analyses confirmed the satisfactory internal reliability of the Polish version of the Somatosensory Amplification Scale (SSAS-PL). The Cronbach’s alpha coefficients of 0.75 for the training sample and 0.77 for the test sample aligned with the generally accepted criteria for psychometric tools (α > 0.70). The average correlations between items (r > 0.23) further supported the internal consistency of the scale. These reliability indices are comparable with or exceed those reported in other adaptations of the SSAS, such as the Turkish adaptation (α = 0.68) [27], French (α = 0.81) [22], and Chinese (α = 0.86 for the nine-item version) [24]. This demonstrates the robust psychometric properties of SSAS-PL as a measurement tool.

An in-depth examination of the discriminatory power of individual test items indicated that item 1 (ang. “When someone coughs near me, I also start coughing”/pol. “Gdy ktoś przy mnie kaszle, mnie też dopada kaszel”) exhibited lower discriminatory power relative to the other items. Its exclusion markedly enhanced the reliability of the scale, as evidenced by an increase in Cronbach’s alpha of 0.77/0.78, mean correlation coefficient of 0.27/0.28, and signal-to-noise ratio of 3.37/3.54. Consequently, it is advisable to exclude this item from the scale’s total score and consider it a buffer.

This observation concerning item 1 is not exclusive to the Polish adaptation. In the Chinese version of the SSAS (SSAS-C), item 1 was similarly excluded due to its low factor loading (0.33), which indicates a weak association with the latent variable [24]. The authors of the Chinese adaptation contended that this item pertains to the influence of others’ bodily processes rather than an individual’s somatic sensitivity, which is central to the somatosensory amplification construct. The consistency in findings between the Polish and Chinese adaptations regarding the problematic nature of item 1 suggests a fundamental conceptual issue with this particular statement in measuring somatosensory amplification. Specifically, item 1 may not align well with the theoretical definition of SSA, which emphasizes an individual’s internal experience and interpretation of their own bodily sensations. Consequently, its removal is not merely a statistical optimization.

### 4.2. Factor Structure

An exploratory factor analysis (EFA) conducted on a population of healthy adults indicated that the two-factor model demonstrated superior fit indices compared with the one-factor model. However, a detailed examination of the two-factor model’s structure revealed challenges in theoretical interpretability, particularly concerning the second factor, which was represented by a single test item. Consequently, the one-factor solution was deemed more theoretically robust and adequately suited for describing the SSAS-PL results.

The original Somatosensory Amplification Scale (SSAS) was developed as a unidimensional instrument intended to assess the general propensity for heightened perception and concern regarding bodily sensations [10]. While most adaptations, including those in French, Chinese, and Iranian contexts, also advocate for a unidimensional structure, some studies proposed models with two or even three factors.

In certain adaptations, such as the Chinese and French versions, a two-factor structure emerged during the analysis, differentiating between extroceptive sensitivity (pertaining to external stimuli, such as noise and smoke) and interoceptive sensitivity (pertaining to internal stimuli, such as heartbeat and hunger). In the Chinese version, the extroceptive factor encompasses items 2, 4, 5, 7, 8, 9, and 10, while the interoceptive factor includes items 1, 3, and 6 [24]. However, in the Chinese adaptation, the correlation between the two factors was exceedingly high (1.03), rendering them statistically indistinguishable and thereby supporting the univariate model. Similarly, in the French adaptation, although two factors were identified, the univariate model demonstrated a superior fit to the data [22].

The recurring pattern observed in numerous cultural adaptations highlights the dilemma between the fit of statistical models and their theoretical consistency. This pattern suggests that the construct of somatosensory amplification, as assessed by SSAS, fundamentally represents a one-dimensional global trend. Despite the theoretical distinctions between exteroceptive and interoceptive sensitivity, when aggregated, the SSAS items predominantly measure a singular, overarching amplification process. A practical implication is that a single overall score derived from the SSAS likely serves as the most reliable and interpretable measure for both clinical and research applications.

The unidimensional structure observed in SSAS-PL is consistent with findings from multiple rigorous cross-cultural adaptations, particularly those employing factor-analytic methods in sufficiently large samples. In the Chinese validation (*N* = 386), confirmatory factor analysis indicated that the nine-item one-factor model, after excluding item 1 due to the low loading (λ = 0.33, R^2^ = 0.10), provided a superior fit: CFI = 0.949, TLI = 0.933, RMSEA = 0.074, and SRMR = 0.0438. A two-factor model yielded an interfactor correlation of r = 1.03, indicating a lack of empirical separation. The reliability indices were robust (Cronbach’s α = 0.86; McDonald’s ω = 0.85), and the test–retest reliability in a subsample (*n* = 133) demonstrated moderate stability (ICC = 0.65). Similarly, the Iranian version (*N* = 240 patients + 30 controls) employed PCA and supported a single-factor solution that explained 29.4% of the variance, with Cronbach’s α = 0.78 and a 1-month test–retest reliability r = 0.80, despite one item showing a low item–total correlation (r = 0.17). In the French adaptation (*N* = 375), both a PCA and CFA were conducted. Although the PCA suggested subtle distinctions between the interoceptive and exteroceptive components, the CFA results favored a unidimensional structure. The internal consistency ranged between α = 0.81 and 0.85, and a strong convergent validity was observed with somatization and trait anxiety measures. In contrast, in the Spanish version [34], a small clinical sample (*N* = 34) was obtained using a PCA alone. They extracted two components, labeled “internal” and “external” amplification, which accounted for 41.1% and 17.2% of the variance, respectively. However, the absence of sample adequacy testing (KMO, Bartlett), a lack of CFA, and reliance on an eigenvalue >1 criterion limits the interpretability. Finally, the German version [35] was validated in a large representative sample (*N* = 2510), where it showed good internal consistency (α = 0.83), but did not include any factor-analytic evaluation; hence, its dimensional structure remains unknown. Collectively, these findings reinforce the validity of the unidimensional SSAS across diverse linguistic and cultural contexts, corroborating its construct coherence when measured using a single total score. The consistent psychometric issues with specific items (notably item 1 in the Chinese study and item 2 in the Farsi validation) suggest fruitful avenues for further item-level analysis (e.g., invariance and IRT) to globally optimize the scale performance.

### 4.3. Convergent and Discriminant Validities

The heterotrait–monotrait (HTMT) analysis demonstrated satisfactory factorial validity and discriminant validity for both the nine-item and eight-item versions of the SSAS-PL. The average variance explained (AVE) was approximately 0.30, and the average percentage of shared variance (ASV) was lower than that of the AVE. This indicates that the variance of the scale exceeded the variance shared with the other constructs. Consequently, the SSAS-PL appeared to measure a unique aspect of somatic experience distinct from health anxiety (SHAI) and psychopathological symptoms (KOOF-58).

The correlation analysis presented in Table 9 indicates that the SSAS-PL score exhibited the strongest positive correlations with somatic symptoms, anxiety disorders, and cognitive disorders, as measured by the KOOF-58 scale. Conversely, the weakest negative correlations were identified with the scales that assessed addiction, lack of entertainment, and suboptimal functioning in occupational and domestic settings.

The observed correlation patterns align with the findings of previous research. Somatosensory amplification is significantly associated with health anxiety disorder (IAD) [36,37], anxiety disorders (ADs) [8], and hypochondria [38]. Associations with alexithymia, particularly concerning difficulties in identifying and describing feelings, are also well-documented (e.g., ref. [2]). The study of the Chinese version of the SSAS-C further demonstrated positive correlations with the SHAI (r = 0.28) and TAS-20 (r = 0.37), thereby confirming the convergent validity of the SSAS-PL with these constructs [22]. Given the definition of SSA as a tendency towards heightened perception of bodily sensations, strong correlations with somatic symptoms are anticipated.

The observed pattern of correlations, wherein the SSAS-PL scores exhibited the strongest associations with somatic symptoms, anxiety, and cognitive disorders, demonstrated weaker or negative correlations with the scales that assessed addiction, lack of entertainment, and poor functioning at work and home, suggesting that somatosensory amplification is not a general indicator of overall distress or impaired functioning. Instead, it represents a more specific phenomenon related to the perception and interpretation of bodily sensations and the accompanying anxiety [39]. This specificity implies that somatosensory amplification is primarily a cognitive phenomenon rather than a manifestation of general negative emotionality [40]. The strong correlations between somatic symptoms and anxiety disorders highlight that SSA is central to how individuals experience and respond to their physical condition, particularly when it results in distress or anxiety. The weaker negative correlations with the functioning scales may indicate that although SSA contributes to subjective distress, it may not directly cause widespread behavioral withdrawal or that the internal focus characteristic of high SSA may indirectly hinder engagement in external activities, leading to perceived functional deficits. 

### 4.4. Clinical Applications of SSAS-PL

The current study’s significant psychometric contribution is the establishment of measurement invariance for the SSAS-PL across both cardiology and psychiatric patient groups. These results bear significant theoretical and practical implications. Theoretically, this suggests that somatosensory amplification constitutes a transdiagnostic psychological construct that is consistently structured and conceptualized across diverse clinical populations. In essence, despite variations in the symptom manifestations (e.g., palpitations in cardiac patients versus anxiety-induced bodily concerns in psychiatric patients), the fundamental propensity to amplify bodily sensations functions through a similar cognitive–perceptual mechanism. The confirmation of configural, metric, and scalar invariances verified that both the factorial structure and item loadings of the SSAS-PL remained stable across diagnostic groups. This provides psychometric validation for comparing total scores and structural relationships with external variables (e.g., anxiety and somatic symptoms) across clinical categories, without the risk of measurement bias. Clinically, these findings endorse the use of the SSAS-PL as a universal screening and assessment instrument for bodily symptom amplification, irrespective of the medical or psychiatric etiology. This suggests that identical interpretative norms and cut-off thresholds can be applied in both cardiology and psychiatry contexts, thereby simplifying clinical integration and facilitating comparative research. Moreover, invariance implies that elevated SSAS-PL scores in either group are likely to reflect authentic differences in the latent construct rather than artificially inflated values due to a scale misfit. This is particularly pertinent in transdiagnostic research and when SSA is employed as a potential treatment target in cognitive–behavioral or psychophysiological interventions. Notably, it opens the possibility of investigating SSA as a shared cognitive–affective process underlying symptom persistence across medical and psychiatric populations.

### 4.5. The Role of SSA in Somatoform and Related Disorders

Somatosensory amplification is recognized as a transdiagnostic factor that significantly contributes to the development and persistence of somatic symptoms that lack comprehensive medical explanations [17]. It is pivotal in the comprehension of somatoform disorders (SSDs) [16]; health anxiety disorders (IADs) [37]; and chronic pain syndromes, such as fibromyalgia (FM) [41].

The present study corroborated the strong correlations of the SSAS-PL with somatic symptoms, health anxiety, and anxiety disorders, consistent with the existing literature. Additionally, SSA is associated with migraine [42] and noncardiac chest pain (NCCP) [18,43].

Barends et al.’s three-year longitudinal study [17] substantiates that SSA is a relatively enduring psychological factor contributing to the persistence of somatic symptoms and diminished functioning. Furthermore, the research conducted by Sayar et al. [21] demonstrated that while antidepressant treatment reduced SSA in patients with depression, SSA levels remained stable in the fibromyalgia group. This finding suggests that SSA is not merely a reflection of general psychological distress, and its variability may be constrained in chronic disorders.

Collectively, these findings indicate that somatosensory amplification (SSA) is not merely a transient manifestation of general psychological distress that varies with mood or responds to general psychotropic medications. Rather, it appears to be a more enduring characteristic that is particularly resistant to modification in chronic somatic conditions, such as fibromyalgia. If SSA is indeed a stable feature that cannot be readily altered by general interventions (e.g., antidepressants), this has significant implications for treatment strategies. This suggests that for patients exhibiting high levels of SSA, especially those with chronic somatic symptom disorders (e.g., fibromyalgia, irritable bowel syndrome, and functional neurological disorder), interventions specifically targeting the mechanisms of somatosensory amplification may be necessary [44]. Such interventions may include the cognitive restructuring of bodily experiences, mindfulness-based cognitive behavioral therapy, or therapies that directly address negative expectations or attentional biases [45,46]. The SSAS-PL, by reliably identifying individuals with high SSA, may serve as a valuable tool for tailoring treatment plans. A study conducted by Ishii [47] revealed that individuals residing in Japan exhibited a higher somatosensory amplification intensity compared with those in the USA, suggesting a more pronounced somatosensory amplification in Eastern cultures. Conversely, a stronger correlation between SSAS and negative emotions was identified among individuals in the USA. These observed differences may be attributed to cultural variations in emotional awareness and the significance of social support and stigma across different ethnic groups [48].

This may guide clinicians towards more specific psychological or psychotherapeutic approaches that directly address the cognitive–perceptual amplification process, potentially leading to more effective and lasting symptom management than generic symptom treatments alone.

Given the properties demonstrated above, the SSAS-PL is a valuable instrument for diagnosing the severity of somatosensory amplification and serves as a screening tool to identify individuals at risk for somatic symptoms and related disorders within primary or psychiatric care settings, particularly when patients exhibit medically unexplained physical symptoms. Furthermore, the SSAS-PL functions as a monitoring tool to evaluate changes in somatosensory enhancement during psychotherapy or pharmacological treatment, especially in cognitive–behavioral interventions that focus on symptom interpretation and interoceptive awareness.

## 5. Conclusions

The current study effectively adapted and validated the Polish version of the Somatosensory Amplification Scale (SSAS-PL), which demonstrated its satisfactory internal reliability and validity. The nine-item version of the scale (items 2–10) is recommended because of its enhanced psychometric properties when item 1 is excluded.

The SSAS-PL had a demonstrated robust measurement invariance across the cardiac and psychiatric patient populations, which underscored its potential utility as a research instrument for evaluating somatosensory amplification in diverse clinical cohorts, as well as the general population.

The SSAS-PL is a valuable instrument for diagnosing, planning, and monitoring therapeutic interventions aimed at the cognitive–emotional processes that underlie somatosensory amplification. This facilitates a more personalized approach for patients with medically unexplained somatic symptoms, and aids in educating patients about the influence of their perceptual style on the symptoms they experience.

Future research should further investigate the construct of somatosensory amplification across a wider range of psychosomatic disorders. It should also endeavor to integrate self-report measures with objective indices of interoception to achieve a more comprehensive understanding of somatosensory amplification. Additionally, there is a need to explore the mechanisms by which high somatosensory amplification develops and persists in greater detail.

## Figures and Tables

**Table 1:** Characteristics of the study sample.

	Healthy *n* = 711 ^1^	Cardiac *n* = 194 ^1^	Psychiatric *n* = 223 ^1^
Sex			
Man	267 (38%)	101 (52%)	47 (21%)
Woman	441 (62%)	93 (48%)	171 (77%)
Other	3 (0.4%)	0 (0%)	5 (2.2%)
Age	40.0 (20.8), 18.0–89.0	64.5 (14.1), 18.0–89.0	25.0 (5.9), 19.0–72.0
Education level			
Middle school	7 (1.0%)	7 (3.6%)	3 (1.4%)
Primary school	18 (2.6%)	19 (9.8%)	4 (1.8%)
Post-secondary school	52 (7.4%)	10 (5.2%)	15 (6.8%)
Secondary school	237 (34%)	84 (43%)	100 (45%)
Higher education	341 (48%)	26 (13%)	94 (43%)
Vocational school	49 (7.0%)	48 (25%)	5 (2.3%)
Place of residence			
Big city	235 (33%)	46 (24%)	136 (62%)
Small town	132 (19%)	79 (41%)	23 (11%)
Medium city	190 (27%)	41 (21%)	42 (19%)
Rural area	147 (21%)	26 (14%)	18 (8.2%)

^1^ *n* (%); Median (mad), Min–Max.

**Table 2:** Characteristics of the training and test samples.

Variable	Training Sample *n* = 426 ^1^	Test Sample *n* = 285 ^1^	*p* ^2^
Sex			0.962
Man	159 (37%)	108 (38%)	
Woman	265 (62%)	176 (62%)	
Other	2 (0.5%)	1 (0.4%)	
Age	42.0 (20.8), 18.0–89.0	38.0 (20.8), 18.0–85.0	0.241
Education level			0.839
Secondary school	5 (1.2%)	2 (0.7%)	
Primary school	10 (2.4%)	8 (2.8%)	
Post-secondary school	32 (7.6%)	20 (7.0%)	
Middle school	138 (33%)	99 (35%)	
Higher education	201 (48%)	140 (49%)	
Vocational school	33 (7.9%)	16 (5.6%)	
Place of residence			0.264
Big city	136 (32%)	99 (35%)	
Small town	88 (21%)	44 (16%)	
Medium town	115 (27%)	75 (27%)	
Rural area	82 (19%)	65 (23%)	

^1^ *n* (%); Median (mad), Min–Max; ^2^ Pearson’s Chi-squared test; Welch Two-Sample *t*-test.

**Table 3:** Summary of reliability analysis results.

Sample	Cronbach’s α	CI95	Mean r	S/N	M (SD)
Training	0.75	0.72–0.79	0.23	2.86	2.86 (0.66)
Test	0.77	0.73–0.81	0.25	3.26	2.82 (0.68)

**Table 4:** Results of the reliability analysis with test item removal in the training sample.

Item	Cronbach’s α	Mean r	S/N
1	0.77	0.27	3.37
2	0.75	0.24	2.81
3	0.74	0.23	2.62
4	0.73	0.22	2.54
5	0.71	0.21	2.34
6	0.74	0.22	2.60
7	0.71	0.20	2.25
8	0.72	0.21	2.39
9	0.71	0.20	2.24
10	0.74	0.23	2.67

**Table 5:** Summary of the results of the internal consistency analysis.

Model	Factor	Cronbach’s α	95% Confidence Interval	Mean r	M	SD	ASE
3-factor							
	MR1	0.73	0.7–0.76	0.31	3.03	0.80	0.02
	MR2	0.47	0.39–0.53	0.23	2.23	0.76	0.03
2-factor							
	MR1	0.71	0.67–0.74	0.26	2.63	0.72	0.02
	MR2	0.60	0.55–0.65	0.34	3.33	0.90	0.03
1-factor							
	Total	0.76	0.73–0.79	0.24	2.84	0.67	0.01

**Table 6:** Summary of the results of the reliability analysis after the omitting items with low discriminatory power.

Sample	Cronbach’s α	CI_95_	Mean r	S/N	M (SD)
Trainig	0.77	0.74–0.8	0.27	3.37	3 (0.72)
Test	0.78	0.74–0.82	0.28	3.54	2.94 (0.73)

**Table 7:** Summary of the fit indices of EFA models to the data in the general population.

Model	χ^2^	df	*p*	RMSEA	CFI	BIC	η^2^
1-factor	119.419	27		0.083 (0.07, 0.1)	0.893	−56.214	0.290
2-factor	48.864	19	<0.001	0.064 (0.04, 0.09)	0.956	−62.730	0.382

*p*—significance of the change in the model fit relative to the previous model; RMSEA—RMSEA (90% confidence interval); η^2^—variance explained by the model.

**Table 8:** Summary of the results of the convergent and discriminant validity analysis using the HTMT method.

Analysis	9 Items	8 Items
Cronbach’s α	0.77	0.76
McDonald’s Ω	0.78	0.77
AVE	0.29	0.30
ASV	0.08	0.08
MSV	0.14	0.17

**Table 9:** Summary of the correlations between the amplification index and the indicators of psychopathological symptoms and health anxiety.

SSA Correlates	r	*p*
Health anxiety	0.302 **	0.002
Poor functioning at work and home	−0.226 *	0.019
Lack of entertainment	−0.233 *	0.015
Poor relationships	0.267 *	0.022
Cognitive disorders	0.469 ***	<0.001
Addictions	−0.169	0.057
Productive symptoms	0.116	0.205
Depressive disorders	0.26 **	0.009
Manic disorders	0.38 **	0.002
Anxiety disorders	0.421 ***	<0.001
Eating disorders	0	0.999
Sleep disorders	0.37 ***	<0.001
Sexual disorders	0.365 **	0.002
Somatic symptoms	0.416 ***	<0.001

* *p* < 0.05, ** *p* < 0.01, *** *p* < 0.001.

**Table 10:** Summary of cross-cultural fit indices for data in the cardiology and psychiatric patient populations.

Model	χ^2^	df	*p*	RMSEA	Lower Range	Upper Range	CFI	BIC
1-factor	93.29	27		0.078	0.06	0.10	0.880	11,876.84
Poland	93.29	27		0.078	0.06	0.10	0.880	11,876.84
China	107.27	34	0.052	0.073	0.06	0.09	0.873	13,012.97
France	103.90	34	0.157	0.071	0.06	0.09	0.879	13,009.59
Iran (Farsi)	78.90	26	<0.001	0.071	0.05	0.09	0.895	11,674.71

*p*—*p*-value for the difference between the current model and the two-factor model in the adult Polish population.

**Table 11:** Summary of the results of the analysis of measurement invariance of the single-factor model in the patient population by type of health disorder.

Invariance	χ^2^	df	*p*	RMSEA	Lower Range	Upper Range	CFI	BIC
Configural	148.26	54.00		0.093	0.07	0.11	0.834	12,000.10
Weak/metric	222.65	71.00	<0.001	0.102	0.09	0.12	0.733	11,972.33
Strong/scalar	206.23	70.00	<0.001	0.098	0.08	0.11	0.760	11,961.92
Latent means	222.65	71.00	<0.001	0.102	0.09	0.12	0.733	11,972.33

## Data Availability

Data available upon request.
